# Global Ischemia of The Cerebellum: The Dark Cerebellar Sign

**DOI:** 10.5334/jbsr.1721

**Published:** 2019-03-11

**Authors:** Cengiz Yılmaz, Ender Alkan, Hasan Erdoğan

**Affiliations:** 1 Aksaray University Education and Research Hospital, TR

**Keywords:** Dark cerebellum, White cerebellum, CT, Trauma

## Case History

**Figure 1:**
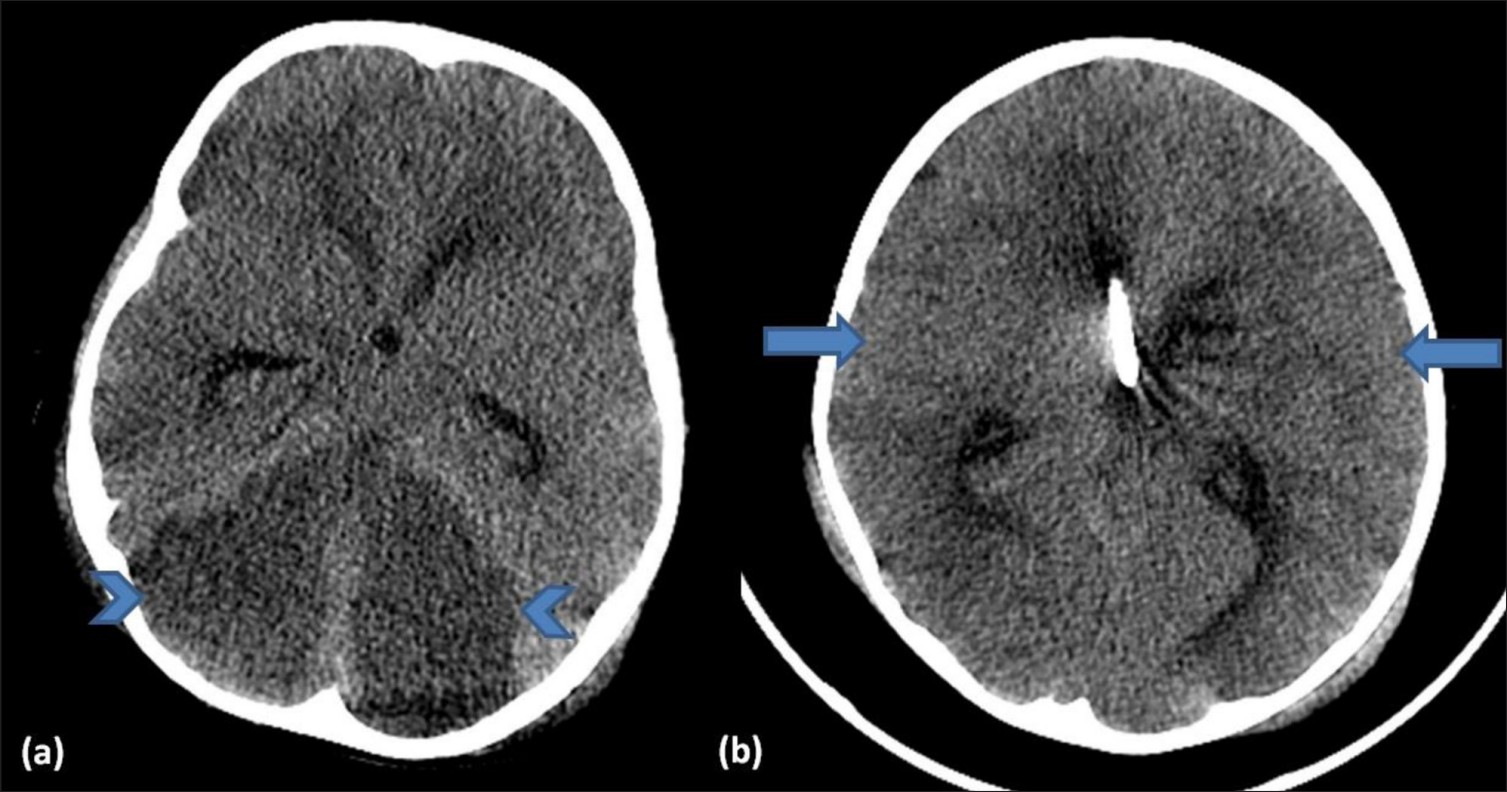


**Figure 2:**
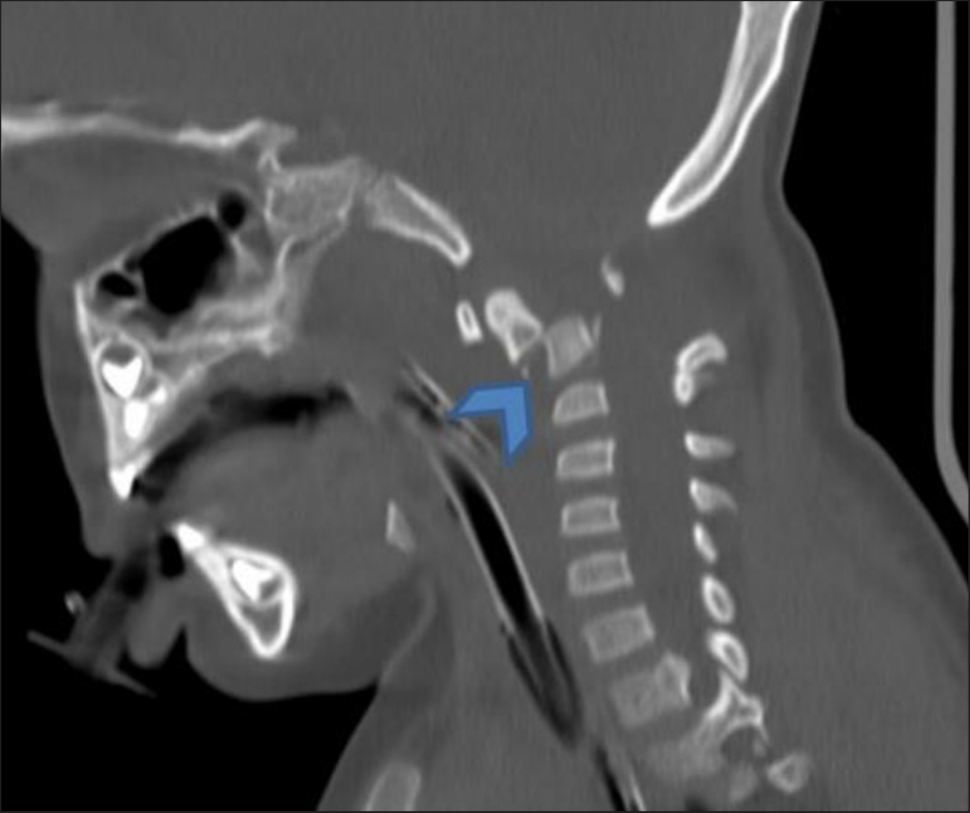


A three-year-old boy with a history of motor vehicle accident presented with loss of consciousness. Non-enhanced cranial computed tomography (CT) (Figure 1) showed diffuse cerebellar hypodensity (open arrows) compared to supratentorial brain parenchyma (arrows). There was also a fracture dislocation of C1-C2 (Figure 2, arrow).

## Comment

We want to present the dark cerebellar sign, which is not known as well as the white cerebellar sign. White cerebellar sign refers to a normal cerebellum and brainstem that appear hyperdense in comparison to the supratentorial brain on non-enhanced CT. The cerebellum and brainstem are believed to be more resistant to hypoxia-ischemia than the supratentorial brain [1]. A diffuse hypodensity of the cerebrum is seen in cases of profound and sustained hypoxia. This may occur in prolonged cardiac arrest, poisoning (carbon monoxide, cyanide, hydrogen sulfide, barbiturate), or as a complication of a severe meningo-encephalitis. On the other hand, the dark cerebellar sign is a much rarer finding and is characterized by a diffuse hypodense cerebellum compared to the normal density of the supratentorial brain parenchyma. The hypodensity of the cerebellum is caused by diffuse parenchymal cerebellar edema and/or infarction [2]. The prognosis is usually very poor. Acute cerebellitis should be in the differential diagnosis of a hypodense, edematous cerebellum [3]. At this situation clinical information may aid differential diagnosis.

In conclusion, dark cerebellar sign refers to diffuse hypodensity of the cerebellum at non-enhanced CT, and develops secondary to diffuse cerebellar ischemia/infarction. In our case diffuse cerebellar infarction probably developed secondary to transection of the vertebral artery.
